# Identifying Periampullary Regions in MRI Images Using Deep Learning

**DOI:** 10.3389/fonc.2021.674579

**Published:** 2021-05-28

**Authors:** Yong Tang, Yingjun Zheng, Xinpei Chen, Weijia Wang, Qingxi Guo, Jian Shu, Jiali Wu, Song Su

**Affiliations:** ^1^ School of Computer Science and Engineering, University of Electronic Science and Technology of China, Chengdu, China; ^2^ Department of General Surgery (Hepatobiliary Surgery), The Affiliated Hospital of Southwest Medical University, Luzhou, China; ^3^ Department of Hepatobiliary Surgery, Deyang People’s Hospital, Deyang, China; ^4^ School of Information and Software Engineering, University of Electronic Science and Technology of China, Chengdu, China; ^5^ Department of Pathology, The Affiliated Hospital of Southwest Medical University, Luzhou, China; ^6^ Department of Radiology, The Affiliated Hospital of Southwest Medical University, Luzhou, China; ^7^ Department of Anesthesiology, The Affiliated Hospital of Southwest Medical University, Luzhou, China

**Keywords:** peri-ampullary cancer, periampullary regions, MRI, deep learning, segmentation

## Abstract

**Background:**

Development and validation of a deep learning method to automatically segment the peri-ampullary (PA) region in magnetic resonance imaging (MRI) images.

**Methods:**

A group of patients with or without periampullary carcinoma (PAC) was included. The PA regions were manually annotated in MRI images by experts. Patients were randomly divided into one training set, one validation set, and one test set. Deep learning methods were developed to automatically segment the PA region in MRI images. The segmentation performance of the methods was compared in the validation set. The model with the highest intersection over union (IoU) was evaluated in the test set.

**Results:**

The deep learning algorithm achieved optimal accuracies in the segmentation of the PA regions in both T1 and T2 MRI images. The value of the IoU was 0.68, 0.68, and 0.64 for T1, T2, and combination of T1 and T2 images, respectively.

**Conclusions:**

Deep learning algorithm is promising with accuracies of concordance with manual human assessment in segmentation of the PA region in MRI images. This automated non-invasive method helps clinicians to identify and locate the PA region using preoperative MRI scanning.

## Introduction

The peri-ampulla (PA) region refers to the area within 2cm of the main papilla of the duodenum, including Vater ampulla, lower segment of common bile duct, opening of pancreatic duct, duodenal papilla and duodenal mucosa nearby (1–4). This region was deep and narrow in the abdomen and has many adjacent organs and blood vessels, so it is difficult to identify this area using conventional imaging examinations. At the same time, the PA region was prone to a series of diseases, including malignant tumors such as periampullary carcinoma (PAC) and benign lesions such as chronic mass pancreatitis, the inflammatory stricture of the lower of common bile duct, or the lower of common bile duct stone etc. (5, 6). The treatment and prognosis of these diseases vary differently, so accurate diagnosis of these disease has important clinical significance. However, the imaging diagnosis of this kind of disease is based on the determination of the specific location of PA region.

So far, among all these modern imaging techniques, magnetic resonance imaging (MRI) is a preferable choice to detect the diseases of the PA region for its advantages of excellent soft-tissue contrast and fewer radiation exposures (5, 7). However, the accuracy and specificity of MRI are still unsatisfying in the diagnosis of the diseases. A study has reported that the specificity of MRI was only 78.26%, while the accuracy was 89.89% in the diagnosis of PAC (5). Similarly, our previous study also found that MRI had only 87% accuracy in detecting PAC (8). For the disease in PA region, misdiagnose will lead to many adverse factors for the follow-up treatment of patients (8, 9). Therefore, it is necessary to further improve the preoperative diagnostic accuracy of the diseases in this special region. Meanwhile, the precise segmentation of PA region is the first and foundation for the accurate diagnosis.

Deep learning is an emerging sub-branch of artificial intelligence that has demonstrated transformative capabilities in many domains (10). Technically, deep learning is a type of neural network with multiple neural layers that is capable of extracting abstract representations of input data like images, videos, time series, natural languages, and texts. Recently, there is a remarkable research advance of applying deep learning in healthcare and clinical medicine (11–13). Deep learning has applications in the analysis of electronic health records, physiological data, and especially in the diagnosis of diseases using medical imaging (14). In the analysis of medical images of MRI, computed tomography (CT), X-ray, microscopy, and other images, deep learning shows promising performance in tasks like classification, segmentation, detection, and registration (15). Recently, considerable literature has grown up in analyzing image segmentation of different human organs using deep learning, such as pancreas (16), liver (17, 18), heart (19), brain (20, 21), etc. However, the PA region remains largely under-explored in medical image analysis based on advanced deep learning algorithms. Though the neural networks have been applied to classify ampullary tumors, the images were taken by endoscopic during operations rather than preoperative and non-invasive MRI or CT scanning (22). To our best knowledge, there is no reported work has been devoted to develop and evaluate deep learning methods to segment the PA region in MRI images.

Therefore, in this study, we presented a deep learning method to automatically segment the PA region in MRI images. We retrospectively collected an MRI image dataset from different types of PA region diseases to train, including PAC and non-PAC patients, so that the PA region could be accurately identified on the MRI image information of different cases. In a training-validation approach, we developed the deep learning method in the training set and validated the performance in the validation set. This would provide a basis for further research on the diagnosis of PAC.

## Materials and Methods

**Figure 1:**
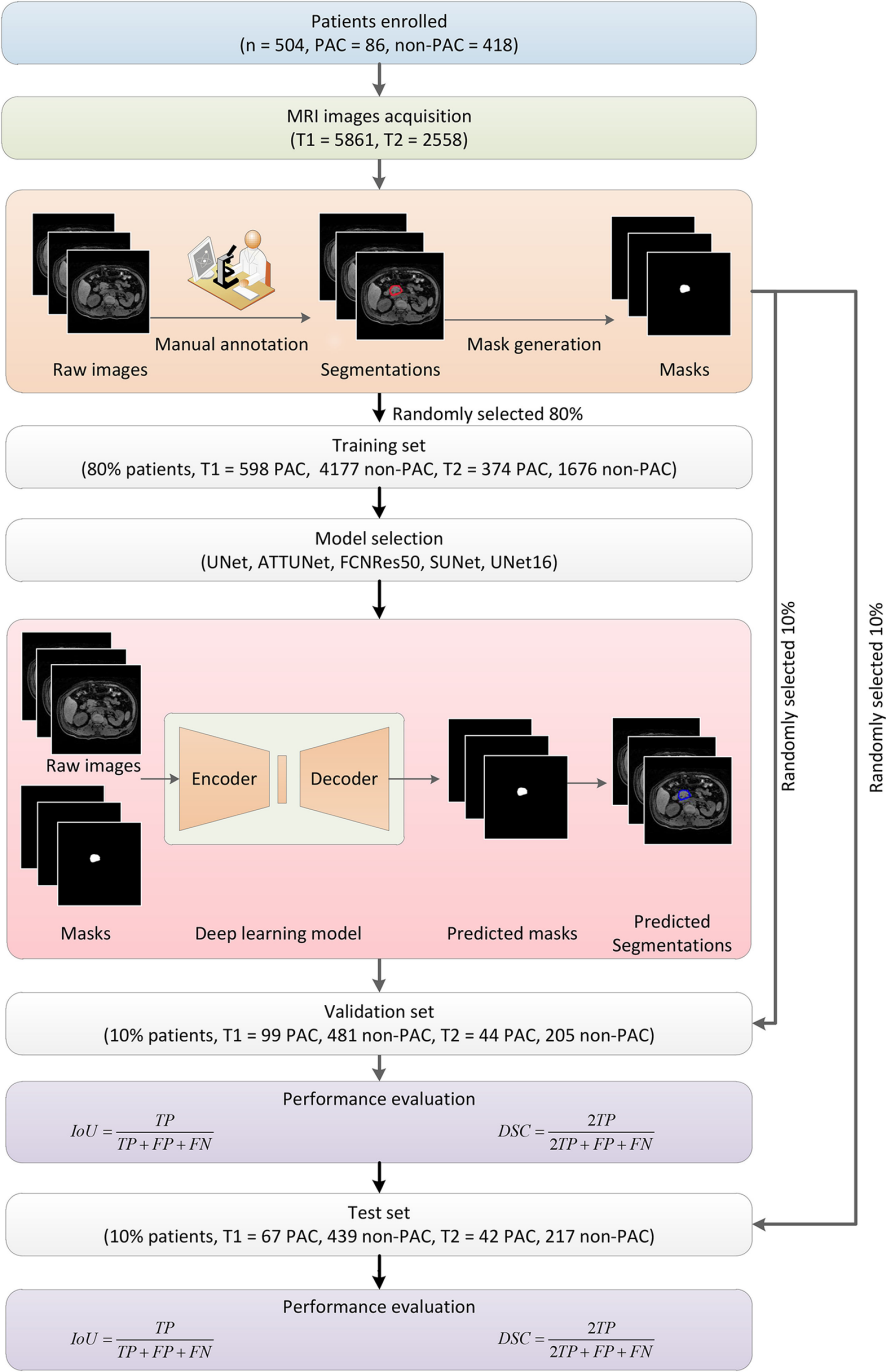
Overall flowchart of this study. First, MRI images were obtained from enrolled patients and manually annotated by experts to obtain the masks for later deep learning algorithm development. The dataset was randomly divided into subsets for algorithm training, validation, and testing, respectively. Five models were developed and evaluated, and the UNet16 and FCNRes50 achieved the best performance.

The overall workflow of this study was illustrated in **Figure 1**. First, patients were included, and the MRI images were obtained. Next, the PA regions were annotated in the MRI images by experts. Based on the raw images and annotation information, the deep learning segmentation algorithms were trained and evaluated in training and validation datasets, respectively. Finally, the performance was summarized and reported.

### Patients Characteristics

This was a retrospective study approved by the Ethics Committee of the Affiliated Hospital of Southwest Medical University (No.KY2020157). A total of 504 patients who underwent MRI examinations in the Department of Hepatobiliary and Pancreatic Surgery of the Affiliated Hospital of Southwest Medical University were included from June 1, 2018 to May 1, 2019. In these people, 86 persons were diagnosed as peri-ampullary carcinoma through pathology after surgery or endoscopy, and the other 418 persons show no peri-ampullary lesion determined by radiologist. All patients underwent MRI examinations. The demographic and clinical characteristics of PAC and non-PAC patients were shown in **Table S1** and **Table S2**, respectively.

### MRI Techniques

After 3-8 hours of fasting, patients were asked to practice their breathing techniques. MRI was performed in all patients with a 3.0-T MR equipment (Philips Achieva, Holland, Netherlands) with a quasar dual gradient system and a 16.0-channel phased-array Torso coil in the supine position. Drinking water or conventional oral medicines were not restricted. The MR scan started with the localization scan, followed by a sensitivity-encoding (SENSE) reference scan. The scanning sequences were as follows: breath-hold axial dual fast field echo (dual FFE) and high spatial resolution isotropic volume exam (THRIVE) T1-weighted imaging (T1WI), respiratory triggered coronal turbo spin echo (TSE) T2-weighted imaging (T2WI), axial fat-suppressed TSE-T2WI, single-shot TSE echo-planar imaging (EPI) diffusion-weighted imaging (DWI), and MR cholangiopancreatography (MRCP). For the dynamic contrast enhancement (DCE)-MRI, axial-THRIVE-T1WI were used. 15mL of contrast agent Gd-DTPA was injected through the antecubital vein at a speed of 2mL/s. DCE-MRI was performed in three phases, including arterial, portal, and delayed phase, and images were collected after 20s, 60s, and 180s, respectively (10). In result, among the 504 patients, 485 patients had THRIVE-T1W images (n = 5,861), and 495 patients had T2 W images (n = 2,558).

### MRI Imaging Analysis

Post-processing of MRI images was performed using the Extended MR Workspace R2.6.3.1 (Philips Healthcare) with the FuncTool package. MRI showed typical PAC imaging manifestations: (1) the mass was nodular or invasive; (2) Tumour parenchyma on T1WI was equal or marginally lower signals; (3) Tumour parenchyma on T2WI was equally or slightly stronger signal; (4) DWI showed high signal intensity; (5) the mass was mild or moderate enhancement after contrast and (6) when MRCP was performed, the bile duct suddenly terminated asymmetrically and expanded proportionally (double-duct signs may occur when the lesion obstructed the ducts (8).

### Pathological Examination

The pathological data from all of the cases were analyzed by two pathologists with more than 15 years of diagnostic experience. The pathologists were blinded to the clinical and imaging findings.

### Image Annotation

First, all MRI images were annotated by two experienced radiologists using in-house software. In the annotation, one radiologist was required to manually draw the outlines of the PA regions in the MRI images. The outline information was used to generate a corresponding mask image in the same size to indicate the segmentation and of the PA region. An expert radiologist reviewed all manual annotations to ensure the quality of the annotations, which served as ground truths to develop and validate deep learning algorithms (23–26).

Among the 504 patients, 485 patients had T1 images (n = 5,861), and 495 patients had T2 images (n = 2,558) were processed separately. We developed algorithms for three cases, namely using only T1, only T2, and combination of T1 and T2. In a cross-validation approach, we first randomly divided the patients into three independent cohorts, namely one training cohort (80%), one validation cohort (10%), and one test cohort (10%). Their images and corresponding annotated mask images were also accordingly grouped into one training set, one validation set, and one test set, respectively. In other words, the MRI images and the corresponding mask images of the training cohort were used to train deep learning algorithms, and those images of the validation and test cohort were later used to select and evaluate the performance of deep learning algorithms.

### Deep Learning Methods

In this study, we developed deep learning algorithms using multiple layers of convolutional neural network (CNN) to automatically segment PA regions in MRI images. CNN is usually utilized to extract hierarchical patterns from images in a feedforward manner. CNN-based deep learning algorithms have achieved remarkable performance in many computer vision applications surpassing human experts (10). In medical image analysis, UNet adopted a two-block structure utilizing multiple layers of CNN (27). More specifically, the architecture consisted of two components. Namely, one encoder transformed the high dimensional input images into low dimensional abstract representations, and one following decoder projected the low dimensional abstract representations back to the high dimensional space by reversing the encoding. Finally, generated images were output with pixel-level label information indicating the PA region. The detailed structures were illustrated in **Figure S1A** for UNet16 and **Figure S1B** for FCNRes50, respectively. In order to systematically investigate the performance of the deep learning approach, in this study, we also considered another four structure variations, namely ATTUNet using the attention gate approach in UNet (27), FCNRes50 using ResNet50 as the downsampling approach FCNRes50 combine residual network and fully convolutional network structures to extract pixel-level information and generate segmentation (28), UNet16 use VGG16 as the downsampling approach (29), and SUNet using SeLu as the nonlinear activation function instead of ReLu.

In the deep learning algorithm training stage, the MRI images of the training cohort were input into the encoder one by one. The output masks generated by the decoder were compared against the corresponding ground truth to calculate the loss function, which indicated the deviations of predicted segmentation. By using the back-propagation technique of stochastic gradient descent optimization, the encoder-decoder structure was continuously optimized to minimize the loss. More technically, the weights between neural network layers were adjusted to improve the capability of segmentations. Once the training started, both the encoder and decoder were all trained together. In this manner, a satisfying deep learning neural network could hopefully be obtained after training with enough training samples. Meanwhile, since the input and output were both images, this deep learning approach enjoyed significant advantages over the conventional image analysis methods by eliminating the exhausting feature engineering or troublesome manual interferences. After the training stage, the trained encoder-decoder structure was used in passive inferences to predict PA regions in MRI images. In inferences, the weights were kept unchanged. In the validation stage, the MRI images of the validation set were input into the neural network, and the corresponding mask images were obtained. The images of the test cohort were used in evaluating the performance of the selected best model. We systematically considered four different variations of the UNet structures and one FCNRes50 structure to seek the best performing deep learning structure. Deep learning algorithms were trained, validated, and tested separately using respective images. The five models were trained, validated, and tested in the dataset contained both T1 and T2 images.

All programs were implemented in Python programming language (version 3.7) with freely available open-source packages, including Opencv-Python (version 4.1.0.25) for image and data processing, Scipy (version 1.2.1) and Numpy (version 1.16.2) for data management, Pytorch (version 1.1) for deep learning framework, Cuda (version 10.1) for graphics processing unit (GPU) support. The training and validation were conducted in a computer installed with an NVIDIA 3090Ti deep learning GPU, 24GB main memory, and Intel(R) Xeon(R) 2.10GHz central processing unit (CPU). It is worth mentioning that the validation task could be done using a conventional personal computer within an acceptable time since the passive inference requires fewer computations.

### Statistical Evaluation of Segmentation

The performance of the segmentation task for the PA region in MRI images was quantitatively evaluated using intersection over union (IoU) and Dice similarity coefficient (DSC). For one PA region instance in an MRI image, the manually annotated ground truth and the deep learning predicted segmentation were compared at pixel-level to see how the two regions overlapped. In general, larger values of IoU and DSC indicated better segmentation accuracies. The average IoU and DSC were calculated based on predictions for all images in the validation set. For simplicity, we used IoU as the main measurement, and the performance of five deep learning structures was ranked according to IoU. The predictions of T1 and T2 MRI images were conducted separately in the same manner.

## Results

### MRI Images

In preparing the training, validation, test datasets, we divided the initial dataset based on patients to ensure that images from a given patient would only appear in one dataset. In result, for T1 images (n = 5,861), the training set included 598 images from 67 PAC patients, and 4,177 images from 322 patients without PAC. The validation set included 99 images from 8 PAC patients, and 418 images from 40 patients without PAC. The test set included 67 images from 8 PAC patients, and 439 images from 40 patients without PAC. For T2 images (n = 2,558), the training set included 374 images from 68 PAC patients, and 1,676 images from 329 patients without PAC. The validation set included 44 images from 8 PAC patients, and 205 images from 41 patients without PAC. The validation set included 42 images from 8 PAC patients, and 217 images from 41 patients without PAC. For the dataset combined T1 and T2 MRI images (n = 8,419). The training set included 959 images from 69 PAC patients, and 5,701 images from 335 patients without PAC. The validation set included 176 images from 9 PAC patients, and 806 images from 42 patients without PAC. The test set included 89 images from 8 PAC patients, and 668 images from 41 patients without PAC.

### Segmentation Performance

**Figure 2:**
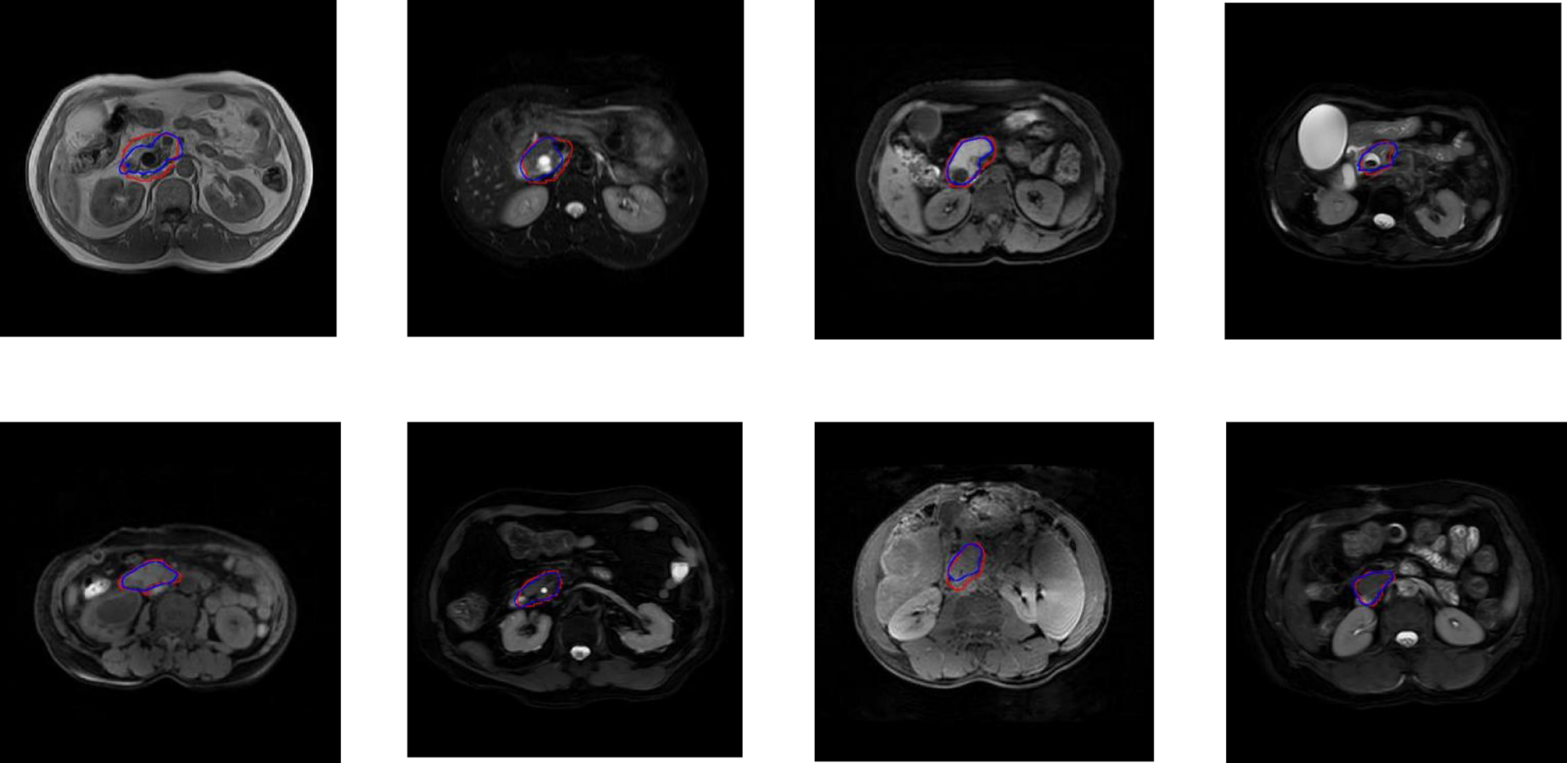
Examples of PA regions of PAC patients (top panel) and PA regions of patients without PAC (bottom panel). The first column were examples of T1 MRI image obtained by UNet16 trained using only T1 images, the second column were examples of T2 MRI image obtained by FCNRes50 trained using only T2 images, the third column were examples of T1 MRI image obtained by UNet16 trained using both T1 and T2 images, and the fourth column were examples of T2 MRI image obtained by UNet16 trained using both T1 and T2 images. Blue, algorithm; red, expert.

For the five segmentation deep learning structures, we followed the same training approach in separated training, validation, and testing. Specifically, each image formed a batch (batch size = 1), and ten rounds were repeated (epoch = 10) to ensure the convergence of the loss. The optimizer of all models is Adam, with a learning rate of 0.0001. The final segmentation performance of all five structures was presented in **Table 1** for T1 images, **Table 2** for T2 images, and **Table 3** for T1 and T2 images, respectively. We found that UNet16 outperformed all the rest structures with the best performance for both of only T1 (IoU = 0.68, DSC = 0.79) and combined T1 and T2 (IoU = 0.64, DSC = 0.74), respectively. The performance of FCNRes50 is better than UNet16 in only T2 (IoU = 0.68, DSC = 0.79) images segmentation. As shown in the tables, the performance of patients with PAC and patients without PAC is calculated, respectively. **Figure 2** demonstrated the segmentation samples obtained by UNet16 for T1 images, FCNRes50 for T2 images, and UNet16 for combined T1 and T2 images. In terms of speed, the algorithms could output the segmentation for a given image within two seconds, which significantly improved the efficiency of image analysis.

**Table 1:** Segmentation performance of deep learning structures in the test T1 images ranked by mean IoU.

Model	IoU			DSC		
	Total	PAC	non-PAC	Total	PAC	non-PAC
**UNet16**	**0.68 ± 0.21**	**0.67 ± 0.18**	**0.69 ± 0.21**	**0.79 ± 0.21**	**0.78 ± 0.17**	**0.79 ± 0.21**
FCNRes50	0.67 ± 0.24	0.65 ± 0.22	0.67 ± 0.24	0.77 ± 0.26	0.76 ± 0.23	0.77 ± 0.26
UNet	0.53 ± 0.33	0.37 ± 0.34	0.55 ± 0.32	0.62 ± 0.36	0.44 ± 0.39	0.64 ± 0.35
SUnet	0.49 ± 0.30	0.40 ± 0.31	0.50 ± 0.30	0.59 ± 0.34	0.50 ± 0.35	0.60 ± 0.33
ATTUnet	0.44 ± 0.32	0.31 ± 0.32	0.46 ± 0.32	0.53 ± 0.37	0.37 ± 0.38	0.55 ± 0.36

UNet16 achieved the best performance.

**Table 2:** Segmentation performance of deep learning structures in the test T2 images ranked by mean IoU.

Model	IoU			DSC		
	Total	PAC	non-PAC	Total	PAC	non-PAC
**FCNRes50**	**0.68 ± 0.20**	**0.66 ± 0.18**	**0.69 ± 0.21**	**0.79 ± 0.21**	**0.78 ± 0.16**	**0.79 ± 0.21**
UNet16	0.67 ± 0.19	0.60 ± 0.21	0.68 ± 0.18	0.78 ± 0.19	0.72 ± 0.21	0.79 ± 0.18
ATTUnet	0.58 ± 0.26	0.51 ±0.29	0.60 ± 0.25	0.69 ± 0.27	0.61 ± 0.32	0.71 ± 0.26
SUnet	0.48 ± 0.25	0.52 ± 0.25	0.47 ± 0.25	0.60 ± 0.28	0.64 ± 0.28	0.59 ± 0.28
UNet	0.40 ± 0.30	0.35 ± 0.29	0.42 ± 0.30	0.50 ± 0.35	0.44 ± 0.34	0.51 ± 0.34

FCNRes50 achieved the best performance.

**Table 3:** Segmentation performance of deep learning structures in the test T1 and T2 images ranked by mean IoU.

Model	IoU			DSC		
	Total	PAC	non-PAC	Total	PAC	non-PAC
**UNet16**	**0.64 ± 0.25**	**0.61 ± 0.18**	**0.65 ± 0.25**	**0.74 ± 0.26**	**0.74 ± 0.18**	**0.74 ± 0.27**
FCNRES50	0.55 ± 0.30	0.47 ± 0.27	0.56 ± 0.30	0.64 ± 0.33	0.59 ± 0.30	0.65 ± 0.33
ATTUnet	0.45 ± 0.34	0.34 ± 0.32	0.46 ± 0.34	0.53 ± 0.38	0.42 ± 0.36	0.54 ± 0.38
SUnet	0.40 ± 0.33	0.28 ± 0.31	0.41 ± 0.33	0.48 ± 0.37	0.34 ± 0.36	0.50 ± 0.37
UNet	0.35 ± 0.35	0.21 ± 0.29	0.37 ± 0.36	0.42 ± 0.40	0.27 ± 0.34	0.43 ± 0.40

UNet16 achieved the best performance.

## Discussion

PAC occurs in 5% of gastrointestinal tumors, and pancreatic cancer is the most common, followed by distal cholangiocarcinoma (2, 30). Pancreatoduodenectomy (PD) was the standard treatment for patients with PAC (31). However, complications such as pancreatic fistula, biliary fistula, infection, and hemorrhage often occur after PD surgery. A previous study has shown that the incidence of postoperative complications of PD may be as high as 30-65% (32). For patients with benign lesions, unnecessary PD surgery could lead to the occurrence of these surgical complications in patients, or even death in some patients. Meanwhile, if malignant lesions are misdiagnosed as benign lesions, it will undoubtedly delay the treatment of patients, resulting in poor prognosis. Due to the anatomical complexity of the periampullary region and less of particular serum markers, the early-accurate diagnose of PAC still remains challenging. Currently, non-invasive diagnostic methods, including ultrasound scan, CT imaging as well as MRI, have been successfully applied to the detection and diagnosis of PAC. One study has reported that the specificity of ultrasound scan was only 52.1%, while the accuracy was 61.61% in the diagnosis of PAC (6). Another study has reported that the specificity of CT was only 16.7%, while the accuracy was 84.4% in the diagnosis of PAC (33). So far, among all these modern imaging techniques, MRI has been reported to be an optimal choice for allowing assessment of periampullary lesions (32). However, there are still limiting factors in the evaluation of the disease using MRI because the PA region is small and the relatively complicated anatomy. Moreover, the tapered area of the distal biliary and pancreatic ducts contain little or no fluid. Physiologic contraction of the sphincter of Oddi also makes it difficult to evaluate the PA region (34). Recently, with the significant development in deep learning and increasing medical needs, artificial intelligence technology has significant advantages in improving the diagnosis of diseases. Therefore, we proposed and developed a deep learning method to automatically segment the PA region in MRI, which could be further extended to future AI-based diagnosis of the disease in PA region using AI, and also facilitate the plan of surgery and endoscopic treatment for clinicians.

In this work, we developed deep learning structures to automatically segment the PA region using MRI T1 and T2 images. Recently, there were abundant reported studies developing AI algorithms for segmentation of abdominal organs or structures including pancreas (16), liver (17, 18), spleen (35, 36), gallbladder (37), kidney (38, 39), the local lesions of stomach (40), etc. However, there is no report of PA region segmentation using AI algorithms. To our best knowledge, this work is the first systematic study of developing and evaluating deep learning approaches for the segmentation of the PA regions in MRI. To evaluate the performance of various deep learning structures, we implemented five algorithms that appeared in deep learning literature, including UNet (27), ATTUNet (41), FCNRes50 (28), UNet16 (29), and SUNet. UNet was the most used deep learning structure in medical image analysis using the encoder and decoder components based on CNN (42). The rest variations improve the UNet structures with attention or replace nonlinear activation functions. This study considered these structures and compared their performance in the same datasets.

In total, 504 patients were included in this study and 5,861 T1 images and 2,558 T2 images were collected. All images were manually annotated by experts to delineate the PA regions in the MRI images. By dividing patients into training and validation cohorts, their images were split into a training set for algorithms training and a validation set for final performance evaluation. As a result, UNet16 achieved the best performance among the five structures with the highest IoU of 0.68 and DSC of 0.79 for T1 images. The model with the best performance for T2 images segmentation is FCNRes50 with an IoU of 0.68 and DSC of 0.79. UNet16 achieved the best performance in the dataset of combined T1 and T2. The IoU is 0.64 and the highest DSC is 0.74 which are not better than the results obtained in the independent T1 or T2 datasets. Therefore, the results showed that UNet16 and FCNRes50 were able to accurately identify the PA region in MRI images.

However, there are still several limitations in this study. First, we only focused on developing an AI to automated localize and segment the PA regions in MRI of PA cancer, but did not make a diagnosis. In the future, we would collect more data and extend the present deep learning framework to classify and diagnose PA cancer. Second, this is a retrospective study from a single hospital, which may inevitably lead to selective bias for the patients. The results need to be validated by prospective and external cohorts. Third, the applied AI technologies in this study are still in rapid evolution with more emerging advanced deep learning algorithms. In the future, it’s necessary to evaluate new deep learning algorithms in PA cancer image analysis to achieve better performance.

In conclusion, we established an MRI image dataset, developed an MRI image data annotation system, established an automatic deep learning the PA region image segmentation model, and realized the location of the PA region.

## Data Availability Statement

The raw data supporting the conclusions of this article will be made available by the authors, without undue reservation.

## Ethics Statement

This was a retrospective study approved by the Ethics Committee of the Affiliated Hospital of Southwest Medical University. Written informed consent for participation was not required for this study in accordance with the national legislation and the institutional requirements. Written informed consent was obtained from the individual(s) for the publication of any potentially identifiable images or data included in this article.

## Author Contributions

YT, YZ, XC, WW, QG, JS, JW, and SS conceived and designed the study, and were responsible for the final decision to submit for publication. All authors contributed to the article and approved the submitted version.

## Funding

This study is supported by the Innovation Method Program of the Ministry of Science and Technology of the People’s Republic of China (M112017IM010700), The Key Research and Development Project of Science & Technology Department of Sichuan Province (20ZDYF1129), The Applied Basic Research Project of Science & Technology Department of Luzhou city (2018-JYJ-45).

## Conflict of Interest

The authors declare that the research was conducted in the absence of any commercial or financial relationships that could be construed as a potential conflict of interest.

## Supplementary Material

Supplementary Figure 1 Schematic diagram of the proposed deep learning algorithms UNet16 **(A)** and FCNRes50 **(B)**. UNet16 is based on an Encoder-Decoder architecture. The encoder was a down-sampling stage, while the decoder was an up-sampling stage. FCNRes50 combine residual network and fully convolutional network structures to extract pixel-level information and generate segmentation. Images and ground truth masks were input into the network to obtain the predicted segmentation.
