# Supplementary material for: Identifying Periampullary Regions in MRI Images Using Deep Learning
Source: Front Oncol. 2021 May 28;11:674579. doi: 10.3389/fonc.2021.674579 (PMC8193851; doi:10.3389/fonc.2021.674579)
Supplement: Supplementary Figure 1 — Schematic diagram of the proposed deep learning algorithms UNet16 (A) and FCNRes50 (B). UNet16 is based on an Encoder-Decoder architecture. The encoder was a down-sampling stage, while the decoder was an up-sampling stage. FCNRes50 combine residual network and fully convolutional network structures to extract pixel-level information and generate segmentation. Images and ground truth masks were input into the network to obtain the predicted segmentation. [file DataSheet_1.docx]

**Supplementary Appendix**

Supplementary Tables S1-S5

Supplementary Figure S1

**Table S1** Clinical characteristics and laboratory findings of included subjects with PAC.

| Variable Median | PAC Value (range) |
| --- | --- |
| Age(years) | 50(25-89) |
| BMI(Kg/m2) | 21.79(19.15-25.26) |
| White Blood Cell (109/L) | 5.84 (2.93–8.52) |
| Neutrophil (109/L) | 3.05 (0.03–6.02) |
| Eosinophil(109/L) | 0.39(0.13–0.63) |
| Hemoglobin (g/L) | 114.54 (90.46–137.16) |
| Platelet (109/L) | 204.76 (101.29–362.61) |
| Total Bilirubin (μmol/L) | 27.00(14.49–47.27) |
| Direct Bilirubin (μmol/L) | 9.98 (3.96–17.65) |
| Total Protein (g/L) | 55.13(21.16–85.78) |
| Albumin (g/L) | 27.58 (14.20–35.92) |
| Alpha Fetoprotein(ng/ml) | 24.49(5.45–43.01) |
| Carbohydrate Antigen19–9 (U/ml) | 83.25 (22.82-173.96) |
| Carbohydrate Antigen 12–5(U/ml) | 39.97(14.81–84.63) |
| Carcinoembryonic Antigen (ng/ml) | 22.07(4.53–42.93) |

**Table S2** Clinical characteristics and laboratory findings of included subjects without PAC.

| Variable Median | Normal Value (range) |
| --- | --- |
| Age (years) | 51 (18-79) |
| BMI(Kg/m2) | 22.42(18.90-26.06) |
| White Blood Cell (109/L) | 7.10(2.25–11.96) |
| Neutrophil (109/L) | 3.39 (1.74–5.37) |
| Eosinophil(109/L) | 0.35 (0.21-0.79) |
| Hemoglobin(g/L) | 134.69(113.54-151.35) |
| Platelet(109/L) | 203.13 (94.47–330.05) |
| Total Bilirubin (μmol/L) | 8.11 (0.53–15.45) |
| Direct Bilirubin (μmol/L) | 5.14 (1.06–9.27) |
| Total Protein (g/L) | 71.36(60.36–102.21) |
| Albumin (g/L) | 41.98(24.78–68.93) |
| Alpha Fetoprotein(ng/ml) | 14.05 (2.54–22.93) |
| Carbohydrate Antigen19–9 (U/ml) | 23.05(4.07-42.71) |
| Carbohydrate Antigen 12–5 (U/ml) | 11.69 (3.90–17.04) |
| Carcinoembryonic Antigen (ng/ml) | 2.92(0.60–5.83) |

**Table S3** Segmentation performance of deep learning structures in T1 images ranked by mean IoU.

|  | | Training set | | Validation set | |
| --- | --- | --- | --- | --- | --- |
|  |  | IoU | DSC | IoU | DSC |
| **UNet16** | Total | **0.80 ± 0.12** | **0.88 ± 0.11** | **0.68 ± 0.16** | **0.80 ± 0.14** |
|  | PAC | **0.78 ± 0.12** | **0.87 ± 0.11** | **0.62 ± 0.12** | **0.76 ± 0.09** |
|  | non-PAC | **0.80 ± 0.12** | **0.88 ± 0.11** | **0.70 ± 0.16** | **0.81 ± 0.15** |
| FCNRes50 | Total | 0.80 ± 0.21 | 0.86 ± 0.21 | 0.62 ± 0.26 | 0.73 ± 0.26 |
|  | PAC | 0.79 ± 0.20 | 0.86 ± 0.19 | 0.50 ± 0.29 | 0.61 ± 0.31 |
|  | non-PAC | 0.80 ± 0.21 | 0.86 ± 0.21 | 0.65 ± 0.24 | 0.75 ± 0.24 |
| SUnet | Total | 0.59 ± 0.29 | 0.68 ± 0.31 | 0.49 ± 0.30 | 0.59 ± 0.34 |
|  | PAC | 0.58 ± 0.29 | 0.67 ± 0.31 | 0.46 ± 0.30 | 0.56 ± 0.35 |
|  | non-PAC | 0.59 ± 0.29 | 0.68 ± 0.31 | 0.49 ± 0.30 | 0.59 ± 0.33 |
| UNet | Total | 0.55 ± 0.34 | 0.63 ± 0.37 | 0.41 ± 0.33 | 0.50 ± 0.36 |
|  | PAC | 0.50 ± 0.35 | 0.58 ± 0.38 | 0.23 ± 0.25 | 0.32 ± 0.30 |
|  | non-PAC | 0.56 ± 0.34 | 0.64 ± 0.37 | 0.45 ± 0.33 | 0.54 ± 0.37 |
| ATTUnet | Total | 0.46 ± 0.34 | 0.54 ± 0.38 | 0.35 ± 0.32 | 0.44 ± 0.37 |
|  | PAC | 0.44 ± 0.33 | 0.52 ± 0.37 | 0.21 ± 0.27 | 0.28 ± 0.32 |
|  | non-PAC | 0.46 ± 0.34 | 0.54 ± 0.38 | 0.38 ± 0.33 | 0.47 ± 0.37 |

**Table S4** Segmentation performance of deep learning structures in T2 images ranked by mean IoU.

|  | | Training set | | Validation set | |
| --- | --- | --- | --- | --- | --- |
|  |  | IoU | DSC | IoU | DSC |
| **FCNRES50** | Total | **0.73 ± 0.21** | **0.82 ± 0.21** | **0.69 ± 0.20** | **0.79 ± 0.20** |
|  | PAC | **0.68 ± 0.25** | **0.77 ± 0.26** | **0.67 ± 0.18** | **0.78 ± 0.17** |
|  | non-PAC | **0.74 ± 0.19** | **0.83 ± 0.19** | **0.69 ± 0.21** | **0.79 ± 0.21** |
| UNet16 | Total | 0.72 ± 0.16 | 0.82 ± 0.16 | 0.68 ± 0.20 | 0.79 ± 0.20 |
|  | PAC | 0.70 ± 0.18 | 0.80 ± 0.18 | 0.68 ± 0.14 | 0.80 ± 0.11 |
|  | non-PAC | 0.73 ± 0.16 | 0.83 ± 0.15 | 0.68 ± 0.21 | 0.78 ± 0.21 |
| ATTUnet | Total | 0.58 ± 0.28 | 0.68 ± 0.30 | 0.58 ± 0.27 | 0.68 ± 0.29 |
|  | PAC | 0.49 ± 0.29 | 0.59 ± 0.33 | 0.52 ± 0.29 | 0.62 ± 0.32 |
|  | non-PAC | 0.60 ± 0.27 | 0.70 ± 0.29 | 0.59 ± 0.27 | 0.70 ± 0.28 |
| SUnet | Total | 0.48 ± 0.26 | 0.60 ± 0.29 | 0.48 ± 0.27 | 0.59 ± 0.30 |
|  | PAC | 0.46 ± 0.26 | 0.57 ± 0.29 | 0.48 ± 0.24 | 0.60 ± 0.27 |
|  | non-PAC | 0.49 ± 0.26 | 0.61 ± 0.29 | 0.47 ± 0.27 | 0.59 ± 0.31 |
| UNet | Total | 0.40 ± 0.31 | 0.49 ± 0.36 | 0.40 ± 0.31 | 0.49 ± 0.36 |
|  | PAC | 0.30 ± 0.30 | 0.39 ± 0.35 | 0.30 ± 0.29 | 0.38 ± 0.34 |
|  | non-PAC | 0.42 ± 0.31 | 0.51 ± 0.35 | 0.42 ± 0.31 | 0.52 ± 0.35 |

**Table S5** Segmentation performance of deep learning structures in both of T1 and T2 images ranked by mean IoU.

|  | | Training set | | Validation set | |
| --- | --- | --- | --- | --- | --- |
|  |  | IoU | DSC | IoU | DSC |
| **UNet16** | Total | **0.78 ± 0.14** | **0.86 ± 0.13** | **0.63 ± 0.24** | **0.74 ± 0.24** |
|  | PAC | **0.76 ± 0.15** | **0.85 ± 0.15** | **0.62 ± 0.18** | **0.75 ± 0.19** |
|  | non-PAC | **0.78 ± 0.13** | **0.87 ± 0.13** | **0.64 ± 0.25** | **0.74 ± 0.25** |
| FCNRes50 | Total | 0.69 ± 0.27 | 0.77 ± 0.28 | 0.57 ± 0.29 | 0.67 ± 0.31 |
|  | PAC | 0.65 ± 0.28 | 0.74 ± 0.29 | 0.58 ± 0.26 | 0.69 ± 0.27 |
|  | non-PAC | 0.69 ± 0.27 | 0.77 ± 0.28 | 0.57 ± 0.29 | 0.67 ± 0.31 |
| ATTUnet | Total | 0.50 ± 0.35 | 0.58 ± 0.38 | 0.45 ± 0.35 | 0.52 ± 0.38 |
|  | PAC | 0.44 ± 0.34 | 0.52 ± 0.37 | 0.33 ± 0.34 | 0.39 ± 0.38 |
|  | non-PAC | 0.51 ± 0.35 | 0.59 ± 0.38 | 0.47 ± .34 | 0.55 ± 0.38 |
| SUnet | Total | 0.44 ± 0.34 | 0.52 ± 0.37 | 0.40 ± 0.33 | 0.48 ± 0.37 |
|  | PAC | 0.40 ± 0.33 | 0.48 ± 0.37 | 0.31 ± 0.32 | 0.38 ± 0.37 |
|  | non-PAC | 0.44 ± 0.34 | 0.53 ± 0.37 | 0.42 ± 0.32 | 0.51 ± 0.36 |
| UNet | Total | 0.42 ± 0.36 | 0.49 ± 0.40 | 0.38 ± 0.35 | 0.45 ± 0.39 |
|  | PAC | 0.34 ± 0.35 | 0.41 ± 0.39 | 0.27 ± 0.32 | 0.33 ± 0.37 |
|  | non-PAC | 0.43 ± 0.36 | 0.50 ± 0.40 | 0.40 ± 0.35 | 0.48 ± 0.39 |

**Figure S1**

**
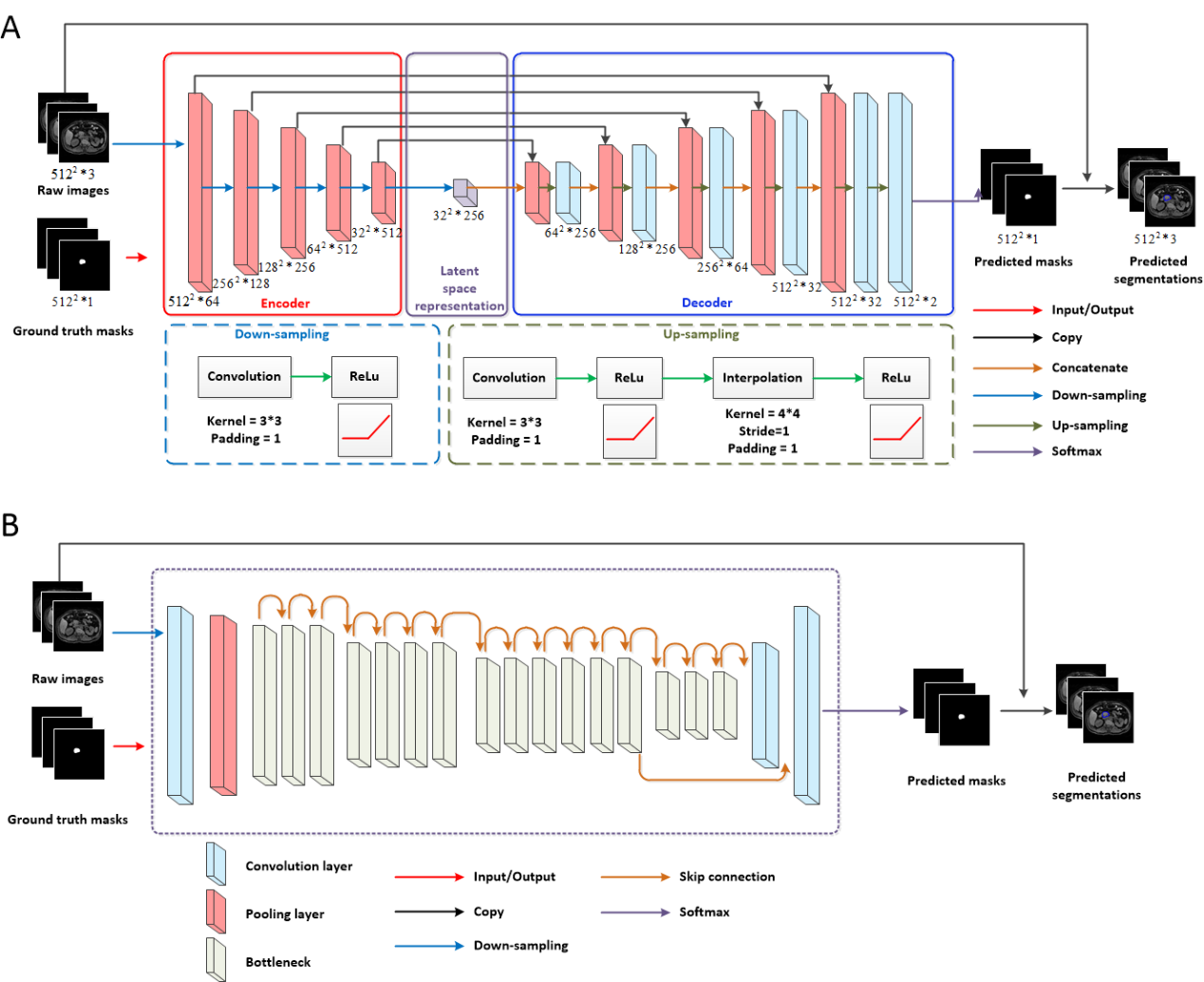
**
